# FHIR-standardized data collection on the clinical rehabilitation pathway of trans-femoral amputation patients

**DOI:** 10.1038/s41597-024-03593-6

**Published:** 2024-07-20

**Authors:** Valerio Antonio Arcobelli, Serena Moscato, Pierpaolo Palumbo, Alberto Marfoglia, Filippo Nardini, Pericle Randi, Angelo Davalli, Antonella Carbonaro, Lorenzo Chiari, Sabato Mellone

**Affiliations:** 1 Department of Electrical, Electronic and Information Engineering “Guglielmo Marconi” – DEI, University of Bologna, Bologna, Italy; 2 Department of Computer Science and Engineering – DISI, University of Bologna, Bologna, Italy; 3 Department of Industrial Engineering – DIN, University of Bologna, Bologna, Italy; 4 Unità operativa di medicina fisica e riabilitazione, INAIL Centro Protesi, Vigorso di Budrio, Italy; 5 Area ricerca e formazione, INAIL Centro Protesi, Vigorso di Budrio, Italy; 6 Health Sciences and Technologies – Interdepartmental Center for Industrial Research (CIRI-SDV), University of Bologna, Bologna, Italy

**Keywords:** Outcomes research, Biomedical engineering, Translational research, Orthopaedics

## Abstract

Lower limb amputation is a medical intervention which causes motor disability and may compromise quality of life. Several factors determine patients’ health outcomes, including an appropriate prosthetic provision and an effective rehabilitation program, necessitating a thorough quantitative observation through different data sources. In this context, the role of interoperability becomes essential, facilitating the reuse of real-world data through the provision of structured and easily accessible databases. This study introduces a comprehensive 10-year dataset encompassing clinical features, mobility measurements, and prosthetic knees of 1006 trans-femoral amputees during 1962 hospital stays for rehabilitation. The dataset is made available in both comma-separated values (CSV) format and HL7 Fast Healthcare Interoperability Resources (FHIR)-based representation, ensuring broad utility and compatibility for researchers and healthcare practitioners. This initiative contributes to advancing community understanding of post-amputation rehabilitation and underscores the significance of interoperability in promoting seamless data sharing for meaningful insights into healthcare outcomes.

## Background & Summary

Major lower limb amputation (i.e., amputation at or proximal to the ankle joint) is a condition affecting about 25 new cases per 100,000 persons each year in European countries^1,2^. It may be the result of different causes, including trauma, vascular diseases, malignancies, and infections^1,3,4^. The amputation invariably affects other physical health aspects of the amputee, produces consequences on their psychological sphere^5^, and causes profound changes in their personal, occupational, and social life^6,7^. People with lower limb amputation commonly exhibit reduced quality of life^8,9^, possibly suffer from pain^10^, residual limb ulcers, gait impairment^11^, and increased risk of falling^12,13^. Anxiety and depression, but also positive psychological transformations, may follow this anatomical loss and life reorganization^5^. An appropriate prosthetic provision and an effective gait and balance rehabilitation program are fundamental for preserving the quality of life^14,15^. Other factors affecting health outcomes and quality of life include age, level of amputation, medication intake, and comorbidities.

While some studies have gathered data on people with amputations and associated clinical details^16,17^, very few datasets have been made publicly available. In 2020, Hood and colleagues have published kinetic and kinematic data from 18 trans-femoral amputees^18^. Furthermore, an early version of the dataset presented here was made available^19^ as part of a study investigating safety of prosthetic knees against falls^20^. A critical need exists to establish a structured database that ensures interoperability and consolidates diverse sources of information with systematic methodologies. Such a database would serve as a potent resource for studying the role of various factors influencing rehabilitation outcomes, prosthetic knee selection, and adverse events.

The standardization of datasets has garnered significant attention in recent years^21,22^, as the adoption of standardized data formats facilitates the sharing and reuse of health data^23^. Within the realm of healthcare data, the Health Level 7 (HL7) organization has developed the Fast Healthcare Interoperability Resources (FHIR) standard^24^, enabling efficient healthcare data exchange with adaptability. Its broad applications, from clinical trials to hospital information systems and public health data streams, significantly contribute to streamlining communications and improving patient care processes^25^. It supports continuity of care at all health system levels, regardless of the software used^26^. The adoption of FHIR as a standard for healthcare data exchange bears several notable advantages in the context of the secondary reuse of real-world data for medical data science, such as cost-effectiveness, increasing quality, and high flexibility of the analysis^27^.

To this end, we introduce the MOTU dataset, a comprehensive dataset of structured data related to rehabilitation hospital stays of patients who have undergone trans-femoral amputation. Data are provided in comma-separated values (CSV) format and in human/machine-readable format employing the FHIR data standard. This dataset is designed to provide valuable insights into the rehabilitation process, contributing to a better understanding of factors influencing patient outcomes and fostering advancements in care for this specific patient population.

This work has been conducted under MOTU and MOTU++, two research projects on trans-femoral amputees and prosthetic devices funded by the Italian National Institute for Insurance against Accidents at Work (INAIL).

## Methods

### Participants

This dataset is the result of a retrospective, observational study conducted at INAIL Prosthesis Centre in Budrio, Italy^20,28^. The INAIL Prosthesis Centre integrates a rehabilitation hospital, a research center, and orthopedic laboratories, assisting every year more than 1500 individuals with amputations resulting from occupational injuries and various other underlying causes.

We included all hospital stays for rehabilitation training of individuals with unilateral trans-femoral amputation or knee-disarticulation, aged 18 years or more, during the period January 2011-May 2020, with signed informed consent by the patient for data treatment for research purposes. We excluded 338 hospital stays because the signed informed consent was either not found (134) or refused (204), representing 14.7% of all the eligible hospital stays.

The study was approved by the Ethics Committee “Area Vasta Emilia Centro” (ref. MOTU 18088, CE AVEC n. 380/2018/OSS/AUSLBO) and complies with the Declaration of Helsinki.

We included 1962 hospital stays of 1006 individuals. Men accounted for 90.9% of all hospitalizations (1784 hospital stays relating to 874 patients); the age range spanned from 18 to 91 years (mean 58, SD 14.4 years). About half of the hospital stays (49.6%, 973) were related to individuals with amputation due to trauma. Slightly more than 20% (21.4%, 420) were hospital stays for rehabilitation training after the first prosthetic provision. The over-representation of men in this cohort is due to two factors: firstly, major lower limb amputations occur more frequently on men than in women^4,29^; secondly, this gender imbalance is even more pronounced for work-related amputations, which in our dataset account for 74% of the total hospital stays^30^.

### Data collection

**Fig. 1:**
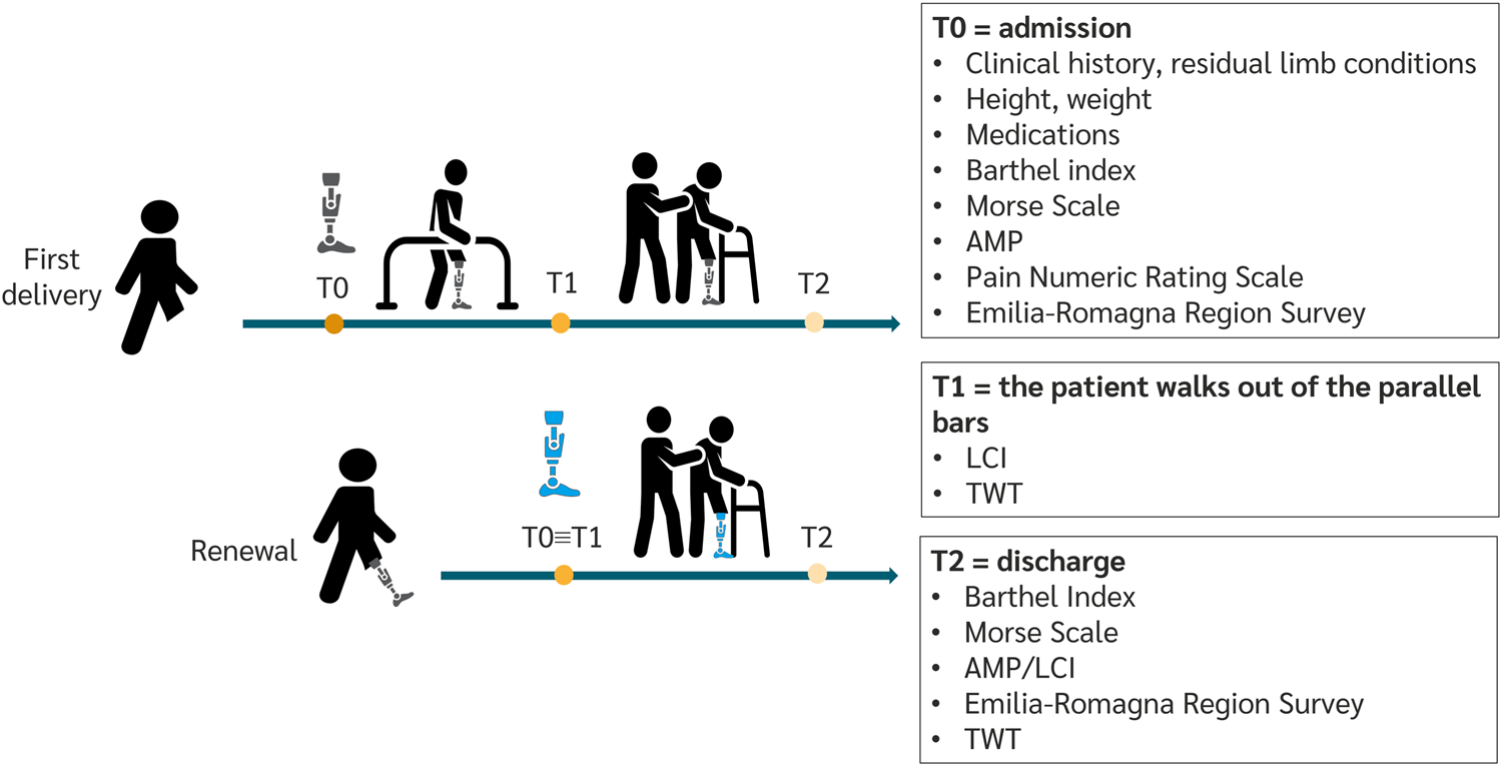
Clinical Pathway. AMP: Amputee Mobility Predictor, LCI: Locomotor Capabilities Index, TWT: Timed Walking Test.

We created the CSV dataset by merging information from patients’ rehabilitation pathway and some external sources. The rehabilitation pathway is schematically shown in Fig. 1. Hospitalizations for rehabilitation training were either for first prosthetic fitting or prosthesis renewal. The former consists of those hospital stays in which the patient received the first prosthetic provision, used during the whole hospitalization at the Centre. The latter represents hospital stays of those patients who returned to the Centre for further rehabilitation after the substitution or significant revision of the prosthesis or its main components (namely the socket, the knee, or the foot).

Three main reference time points can be identified within each hospital stay:

- T0. It represents the patient’s admission to the Centre. At this time, a comprehensive assessment of the patient’s clinical and functional status is conducted to ascertain baseline conditions and delineate a personalized rehabilitation pathway;
- T1. It denotes the exit from the parallel bars during the hospital stays for first prosthetic fitting, and it corresponds to T0 in hospital stays for prosthesis renewal;
- T2. It is the discharge, the moment when the patient ends his/her hospitalization.

**Fig. 2:**
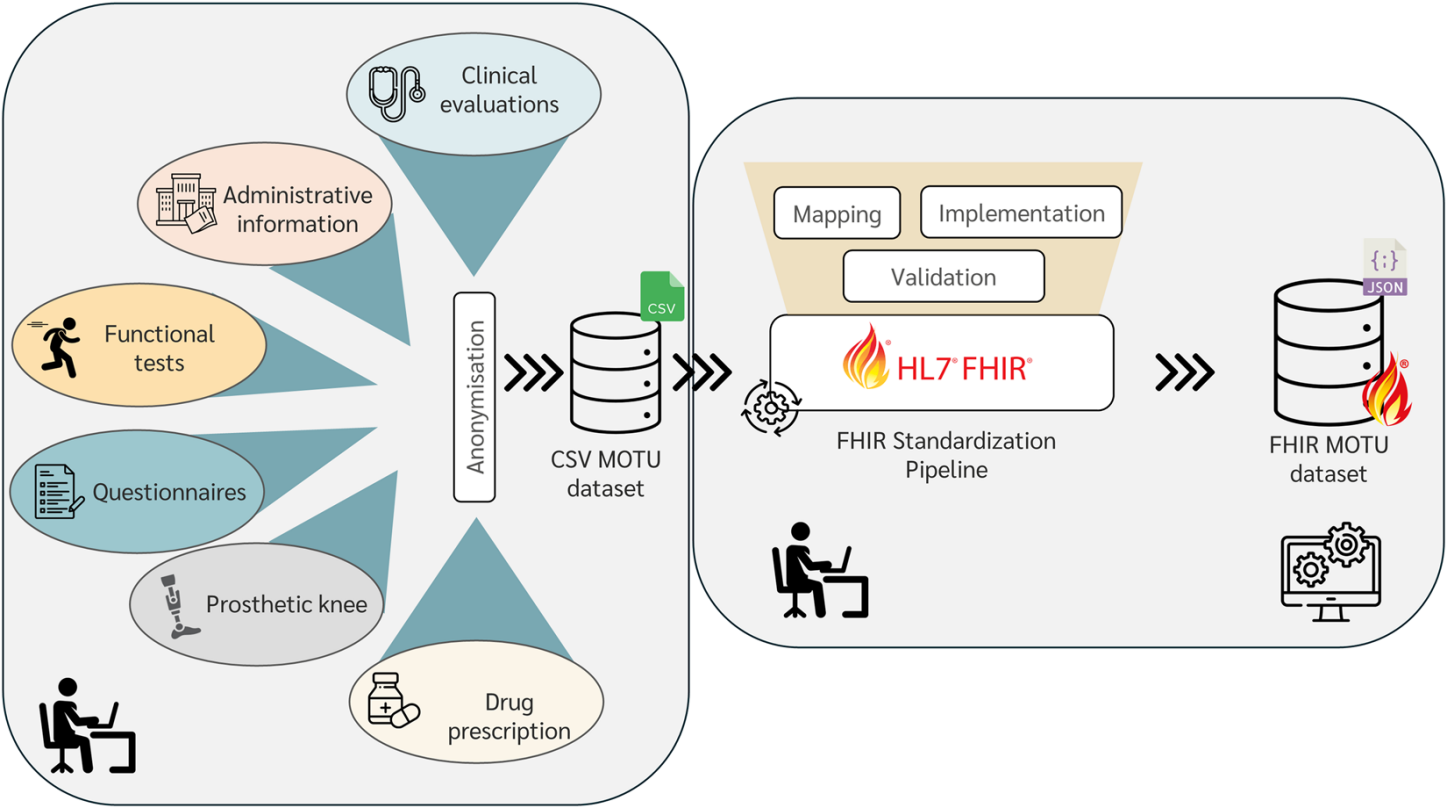
Operational pipeline. Different data sources related to different areas (clinical evaluations, administrative information, functional tests, questionnaires, prosthetic knee, and drug prescription) were merged and anonymised to create the CSV dataset. We then mapped the variable of the CSV dataset into corresponding FHIR resources, identified appropriate coding systems, and validated the FHIR resources (standardization pipeline), thus creating the FHIR MOTU dataset.

The MOTU dataset covers the following areas (Fig. 2):

- *Clinical evaluations*. They consist of the assessment of anthropometric measures, the reason for the current hospitalization, the patient’s medical history, information regarding the amputation (*i.e*., amputation date, side, cause, and the residual limb length), a pain evaluation, and falls occurring during the hospital stay. A fall was defined as a “sudden, unintentional, and unexpected descent from upright, seated, or clinostatic position”^31^. Each fall was registered following the Italian Ministry of Health’s recommendation on fall prevention and management in healthcare settings^32^;
- *Administrative information*. It reports information about the third-party payer of the hospitalization: whether the INAIL institute (for work-related injuries), the national health system, or the patient him/herself;
- *Functional tests*. The patients were assessed for their functional abilities with the following tests:
  - the 10-m Timed Walking Test (TWT)^33^ was executed at T1 and T2. Each TWT was executed twice, at comfortable gait speed, over a 14-m clear path with four marks at 0, 2, 12, and 14 meters. A physiotherapist recorded the time and the number of steps taken between the two intermediate marks.
  - The Amputee Mobility Predictor (AMP)^34^ was executed at T0 and T2 since 2016. It measures the functional mobility of a person with lower-limb amputation, including gait and several tasks related to static and dynamic equilibrium. It was administered without wearing the prosthesis (AMPnoPRO) on amputees at T0 for their first prosthetic fitting and while using the prosthesis (AMPPRO) in all other cases;
- *Questionnaires*. Patients were asked to respond to the following questionnaires:
  - Barthel index^35^, assessing the independence level in activity of daily living. It was administered at T0, during the hospital stay, and at T2;
  - Morse scale^36^, used to determine inpatients’ fall risk. It was administered at T0 and T2 until 2017;
  - Emilia-Romagna Region (ERR) Survey for multifactorial risk assessment for falls in the hospital, developed by the Emilia-Romagna Region in the “Falls prevention in older people” plan^37^. It was administered in T0 and T2 since 2017, substituting the Morse scale;
  - Locomotor Capability Index with 5-level ordinal scale (LCI-5)^38^, specifically designed and validated on persons with lower-limb amputation, assesses the patient’s perceived ability to carry out 14 locomotor activities of daily living while wearing a prosthesis. It was administered at T1 and T2. It has been substituted by AMP in 2016 because it exhibited a ceiling effect in patients with high functional abilities and did not distinguish among different types of walking aid used.
- *Prosthetic knee*. We collected information about the prosthetic knee used by each patient at each hospital stay. We further recorded in the MOTU dataset some characteristics of the prosthetic knees as reported in the manufacturers’ websites. Based on these characteristics, we also categorized the prosthetic knees into four groups: (i) prosthetic knees used in locked configuration during walking (LK); (ii) articulating mechanical knees without fluid control (AMK); (iii) non-electronic, fluid-controlled knees (FK); and (iv) microprocessor-controlled knees (MPK).
- *Drug prescription*. We collected information on all the drugs administered to each patient during the hospital stays. We mapped the Italian drug trade names to the related Anatomical Therapeutic Chemical (ATC) code^39^ according to their main active ingredient. This mapping was supported by tables made available by the Italian Medicines Agency (AIFA^40^) and by manual search over DrugBank^41^.

### Anonymization

We generated an anonymous ID for each patient in the dataset. We excluded from the dataset any variable containing name, surname, fiscal code of the patients or with text in natural language. We further shifted each patient’s dates by a random number of days between −90 and +90 to make deidentification stronger while allowing data analyses on secular trends.

### FHIR standardization

Two experts proficient in HL7 FHIR R4 independently annotated the variables of the CSV dataset into corresponding FHIR resources (Fig. 2). This phase was followed by a discussion on resolving discrepancies in the mapping process, leading to establishing an agreed resource mapping. Once all variables were successfully mapped, per HL7 recommendations, we identified appropriate coding systems for some of the MOTU dataset variables. We adopted a modular template approach using MatchBox presented in a previous study^42^ and particularly regarding on questionnaires we designed a template to aggregate all the item scores from each scale. Finally, we defined customized Search Parameters and used those outlined in the FHIR specification to map data related to counting instances or detecting the presence or absence of specific variables. These resources are defined in terms of FHIRPath expressions and, upon integration into the FHIR server, can be leveraged within the FHIR Search application programming interface (API).

## Data Records

The CSV^43^ and FHIRed^44^ datasets are available for access and utilization at the Zenodo Repository.

### CSV Dataset organization

The CSV dataset consists of five different tables, and its overall structure is presented in Table 1.

- *Patient*. This table relates the 1006 anonymous ID to the patient’s birth date and sex.
- *HospitalStay*. This table contains information about the hospital stays for rehabilitation training, including clinical evaluations, administrative information, and outcomes from functional tests and questionnaires, whose detailed information are provided in Table 2. Each row of this table represents a different hospital stay, identified by the unique combination of the anonymous patient ID and admission date, counting for a total number of 1962 entries.
- *ProstheticKnee*. This table provides technical features for about 40 distinct prosthetic knee models employed by the patients during their hospital stays. Technical features include name of the manufacturer, possibility to manually lock the knee, polycentric design, hydraulic or pneumatic control, electronic (microprocessor) control, knee category (i.e., AMK, FK, LK, or MPK), weight of the device, maximum weight allowed for the patient, patient activity level, and link to the manufacturer’s webpage.
- *Fall*. This table presents 146 entries on information about falls experienced by the patients during their hospital stays: date, whether the patient was wearing a prosthetic knee or not, reported injuries, whether it was a near fall^45^, or the activity carried out at the moment of falling.
- *Drug*. This table lists 3032 entries about all the drugs administered to the patients during their hospital stays. Each hospital stay is identified by the patient anonymous ID and the hospital admission date. Each drug is identified by its trade name in Italy and is associated to the ATC code of its main active ingredient.

**Table 1:** Data descriptor of the MOTU dataset.

Retrospective MOTU dataset				Retrospective MOTU dataset on FHIR	
Table	Label	Description	Type	Result	FHIR Mapping
Patient	AnonymousID	Patient identifier	Integer	Mapped	Patient.identifier
	Sex	Patient gender (i.e. M = Male and F = Female)	Categorical	Mapped	Patient.gender
	BirthDate	Patient birthday	Date	Mapped	Patient.birthDate
Hospital Stay	AnonymousID	Patient identifier	Integer	Mapped	Patient.identifier
	AdmissionDate	Start date of the hospital stay	Date	Mapped	Encounter.startDate
	DischargeDate	End date of the hospital stay	Date	Mapped	Encounter.endDate
	Consent	Flag for patient consent (i.e. 1 = true, 0 = false)	Categorical	Mapped	Consent.status
	ThirdPayer	Payer for the hospital stay (i.e., INAIL, ASL, Private)	Categorical	Mapped	Accounte.coverage– > Coverage^*a*^
	FirstdeliveryRenewal	If the hospital stay consists in either a first prosthetic provision or a renewal (i.e., FirstDeliv, Renewal)	Categorical	Mapped	Encounter.hospitalization.reAdmission
	LengthOfStay	Length of the hospital stay (i.e., number of days)	Integer	Mapped	Encounter.length
	RehabGoal	Rehabilitation goal reached at discharge (i.e. free walk = gait without aids, aid1 = gait with one single aid, aid2 = gait with two aids, walker = gait with walker)	Categorical	Mapped	Careplan– > to Goal.description^*a*^
	AmputationDate	Amputation Date	Date	Mapped	Procedure.occurence_x_
	AmputationSide	Amptuation Side (i.e. L = left or R = right)	Categorical	Mapped	Procedure.bodySite
	AmputationCause	Cause of amputation (i.e. cancer, congenital, infectious, traumatic, vascular)	Categorical	Mapped	Procedure.reason
	StumpLength	Length of the residual limb (i.e. proximal third, distal third, medial third, Knee disarticulation)	Categorical	Not Mapped	
	Height	Patient height (m)	Decimal	Mapped	Observation[1].component[1].value
	Weight	Patient weight (without prosthesis) (kg)	Decimal	Mapped	Observation[1].component[2].value
	NComorbidities	Number of comorbidities	Integer	Derived	
	DrugAntipsychotics	If patient takes antipsychotics (i.e., 1 = yes, 0 = no)	Categorical	Derived	FHIR Search Query on MedicalStatement
	DrugAntidepressants	If patient takes antidepressants (i.e., 1 = yes, 0 = no)	Categorical	Derived	FHIR Search Query on MedicalStatement
	DrugBenzodiazepines	If patient takes benzodiazepines (i.e., 1 = yes, 0 = no)	Categorical	Derived	FHIR Search Query on MedicalStatement
	DrugLoopDiuretics	If patient takes Loop Diuretics (i.e., 1 = yes, 0 = no)	Categorical	Derived	FHIR Search Query on MedicalStatement
	DrugBetaBlockingAgents	If patient takes Betablocking agents (i.e., 1 = yes, 0 = no)	Categorical	Derived	FHIR Search Query on MedicalStatement
	DrugOpioids	If patient takes opioids (i.e., 1 = yes, 0 = no)	Categorical	Derived	FHIR Search Query on MedicalStatement
	DrugAntiepileptics	if patient takes antiepileptics (i.e., 1 = yes, 0 = no)	Categorical	Derived	FHIR Search Query on MedicalStatement
	NDrugs	Number of drugs per hospital stay	Integer	Derived	Operation on MedicationStatement resource
	PainControlateralLimb	0–10 Numerical Rating Scale of Pain	Integer	Mapped	Observation[2].component[1].value
	PainControlateralKnee		Integer	Mapped	Observation[2].component[2].value
	PainBack		Integer	Mapped	Observation[2].component[3].value
	PainStump		Integer	Mapped	Observation[2].component[4].value
	PainPhantomLimb		Integer	Mapped	Observation[2].component[5].value
	Locomotor Capabilities Index (LCI-5)	Patient’s perceived capability to perform 14 different locomotor activities while wearing a prosthesis	Questionnaire	Mapped	Questionnaire
	Amputee Mobility Predictor - Wearing the prosthesis	Assessment of patient’s functional capabilities without prosthesis and to predict his/her ability to ambulate. It may be done while wearing a prosthesis (PRO, 13 items), or not (noPRO, 12 items)	Questionnaire	Mapped	Questionnaire
	Morse Fall Scale	Assessment of patient’s likelihood of falling. It is composed of 6 items	Questionnaire	Mapped	Questionnaire
	10-meter Timed Walking Test (TWT)	Test to assess locomotor capacity in clinical and research settings. Outcome measures are time taken to complete the test and the number of steps	Clinical Test	Mapped	Observation[3].component[1,2]
	Barthel Index	Assessment of patient’s degree of independence in activities of daily living. It is composed of 10 items	Questionnaire	Mapped	Questionnaire
	Emilia-Romagna Region (ERR) Survey	Fall risk assessment, composed of 6 items	Questionnaire	Mapped	Questionnaire
	HFall	If the patient had or not a past history of falls (i.e., 1 = yes, 0 = no)	Integer	Not Mapped	
	DrugCardio	If patient takes cardiovascular medication (i.e., 1 = yes, 0 = no)	Categorical	Derived	FHIR Search Query on “MedicationStatement” resource
	DrugPsychotropic	If patient takes psychotropic medication (i.e., 1 = yes, 0 = no)	Categorical	Derived	FHIR Search Query on “MedicationStatement” resource
	ThreeMoreDrugsExceptCardioPsycho	If the patient takes three more drugs, except for cardiovascular and psychotropic medications (i.e., 1 = yes, 0 = no)	Categorical	Derived	FHIR Search Query on “MedicationStatement” resource
	Diabetes	If the patient has the diabetes or not (i.e., True or False)	Categorical	Mapped	Condition
	KneeModel_string	Commercial name of knee model	String	Mapped	DeviceDefinition.identifier
ProstheticKnee	ProstheticKnee	Commercial name of knee model	String	Mapped	DeviceDefinition.deviceName
	Manufacturer	Manufacturer of the prosthetic knee	String	Mapped	DeviceDefinition.manufacturer.manufacturerString
	ManualLock	If the prosthetic knee can be manually locked or not (i.e., 1 = yes, 0 = no)	Categorical	Mapped	DeviceDefinition.property
	Polycentric	If the prosthetic knee is polycentric or not (i.e., 1 = yes, 0 = no)	Categorical	Mapped	DeviceDefinition.property
	HydraulicPneumaticControl	Ifr the prosthetic knee has a hydraulic or pneumatic control or not (i.e., 1 = yes, 0 = no)	Categorical	Mapped	DeviceDefinition.property
	MPK	If the prosthetic knee is microprocessor-controlled or not (i.e., 1 = yes, 0 = no)	Categorical	Mapped	DeviceDefinition.property
	Weight	Prosthetic knee weight (kg)	Decimal	Mapped	DeviceDefinition.property
	PatientMaximumWeight	Maximum weight of the person that can be supported by the prosthesis (kg)	Integer	Mapped	DeviceDefinition.property
	PatientActivityLevel	Activity level of the patient appropriate for that specifici prosthetic knee (expressed either in terms of K-level or in natural language)	Categorical	Mapped	DeviceDefinition.property
	Link	URL to prosthetic knee website	String	Mapped	DeviceDefinition.contact
	Quality	Knee category (i.e., AMK, FK, LF, MPK)	Categorical	Mapped	DeviceDefinition.property
Fall	AnonymousID	Patient identifier	Integer	Mapped	AdverseEvent.Subject– > Patient.identifier^*a*^
	AdmissionDate	Start date of the hospital stay	Date	Mapped	AdverseEvent.Encounter– > Encounter.startDate^*a*^
	FallDate	When the fall occurred	Date	Mapped	AdverseEvent.occurrence
	WearingProsthesis	If the patient was wearing a prosthesis or not during the fall (i.e., 1 = yes, 0 = no)	Categorical	Mapped	AdverseEvent.referenceDocument– > DocumentReference.description^*a*^
	Injury	Injuries reported by the patient after the fall (reported in natural language)	String	Mapped	AdverseEvent.referenceDocument– > DocumentReference.description^*a*^
	NearFall	If the patient experienced a near fall or not (i.e., 1 = yes, 0 = no)	Categorical	Mapped	AdverseEvent.referenceDocument– > DocumentReference.description^*a*^
	FallActivity	What the patient was doing when fell (reported in natural language)	String	Mapped	AdverseEvent.referenceDocument– > DocumentReference.description^*a*^
Drug	AnonymousID	Patient identifier	Integer	Mapped	MedicationRequest.Subject– > Patient.identifier^*a*^
	AdmissionDate	Start date of the hospital stay	Date	Mapped	MedicationRequest.Encounter– > Encounter.startDate
	DrugTradename	Trade name of the medication	String	Mapped	MedicationRequest.identifier
	ATC	Anatomical Therapeutic Chemica (ATC) code	Integer	Mapped	MedicationRequest.code

^a^Resource reference attribute (“– >” notation).

**Table 2:** Description of questionnaire entries.

Retrospective MOTU dataset	Label	Description	Range/Unit of measure	Type	Retrospective MOTU dataset on FHIR	
Questionnaire					Result	FHIR Mapping
Locomotor Capabilities Index (LCI-5)	LCIInitialDate	LCI administration date at admission		Date	Mapped	Questionnaire
	LCIInitialScore	LCI score at admission	0–56	Integer	Mapped	Questionnaire
	LCIDischargeDate	LCI administration date at discharge		Date	Mapped	Questionnaire
	LCIDischargeScore	LCI score at discharge	0–56	Integer	Mapped	Questionnaire
Amputee Mobility Predictor (AMP)	AMPAdmissionDate	AMP administration date at admission		Date	Mapped	Questionnaire
	AMPAdmissionProNopro	Use of prosthesis (AMPPRO) or without (AMPnoPRO) in fulfilling the questionnaire at admission		Categorical	Mapped	Questionnaire
	AMPAdmissionScore	AMP general score at admission	AMPnopro (0–43) AMPPro (0–47)	Integer	Mapped	Questionnaire
	KlevelAdmission	K-level value at admission		Categorical	Mapped	Questionnaire
	AMPDischargeDate	AMP administration date at discharge		Date	Mapped	Questionnaire
	AMPDischargeProNopro	Use of prosthesis (AMPPRO) or without (AMPnoPRO) in fulfilling the questionnaire at discharge		Categorical	Mapped	Questionnaire
	AMPDischargeScore	AMP general score at discharge	AMPnopro (0–43) AMPPro (0–47)	Integer	Mapped	Questionnaire
	KlevelDischarge	K-level value at discharge		Categorical	Mapped	Questionnaire
Morse	MorseAdmissionDate	Morse administration date at admission		Date	Mapped	Questionnaire
	MorseAdmissionHfall	History of falling at admission	0 = no 25 = fall in previous three months	Integer	Mapped	Questionnaire
	MorseAdmissionPathologies	Secondary Pathologies Assessment at admission	0 = no 15 = presence of secondary at risk diagnosis (e.g. diabetes, cardiovascular disease, hypertension, use of sedatives, antiepileptics, diuretics)	Integer	Mapped	Questionnaire
	MorseAdmissionMobility	Mobility assessment (e.g. walking aid, rollator, crutches, etc) at admission	0 = no walking aids/wheelchair/bedrest 15 = walking aids 30 = walk holding on to the furniture	Integer	Mapped	Questionnaire
	MorseAdmissionEndovenous	Adoption of intravenous therapy/heparin lock at admission	0 = no 20 = yes	Integer	Mapped	Questionnaire
	MorseAdmissionTransfer	Gait assessment (e.g. curve posture, low balance, abnormal, etc.) at admission	0 = normal gait/bedrest/immobile 10 = imperfect gait 20 = unsafe gait	Integer	Mapped	Questionnaire
	MorseAdmissionMental	Mental status assessment at admission	0 = conscious of their abilities 15 = unconscious of their abilities	Integer	Mapped	Questionnaire
	MorseAdmissionTotalScore	Overall Morse score at admission	0–125	Integer	Mapped	Questionnaire
	MorseDischargeDate	Morse administration date at discharge		Date	Mapped	Questionnaire
	MorseDischargeHfall	History of falling at discharge	0 = no 25 = fall in previous three months or during the hospital stay	Integer	Mapped	Questionnaire
	MorseDischargePathologies	Secondary Pathologies Assessment at discharge	0 = no 15 = presence of secondary at risk diagnosis (e.g. diabetes, cardiovascular disease, hypertension, use of sedatives, antiepileptics, diuretics)	Integer	Mapped	Questionnaire
	MorseDischargeMobility	Mobility assessment (e.g. walking aid, rollator, crutches, etc) at discharge	0 = no walking aids/wheelchair/bedrest 15 = walking aids 30 = walk holding on to the furniture	Integer	Mapped	Questionnaire
	MorseDischargeEndovenous	Adoption of intravenous therapy/heparin lock at discharge	0 = no 20 = yes	Integer	Mapped	Questionnaire
	MorseDischargeTransfer	Gait assessment (e.g. curve posture, low balance, abnormal, etc.) at discharge	0 = normal gait/bedrest/immobile 10 = imperfect gait 20 = unsafe gait	Integer	Mapped	Questionnaire
	MorseDischargeMental	Mental status assessment at discharge	0 = conscious of their abilities 15 = unconscious of their abilities	Integer	Mapped	Questionnaire
	MorseDischargeTotalScore	Overall Morse score at discharge	0–125	Integer	Mapped	Questionnaire
	MorseChangeDate	Morse administration date at change		Date	Mapped	Questionnaire
	MorseChangeHfall	History of falling at change	0 = no 25 = fall in previous three months or during the hospital stay	Integer	Mapped	Questionnaire
	MorseChangePathologies	Secondary Pathologies Assessment at change	0 = no 15 = presence of secondary at risk diagnosis (e.g. diabetes, cardiovascular disease, hypertension, use of sedatives, antiepileptics, diuretics)	Integer	Mapped	Questionnaire
	MorseChangeMobility	Mobility assessment (e.g. walking aid, rollator, crutches, etc) at change	0 = no walking aids/wheelchair/bedrest 15 = walking aids 30 = walk holding on to the furniture	Integer	Mapped	Questionnaire
	MorseChangeEndovenous	Adoption of intravenous therapy/heparin lock at change	0 = no 20 = yes	Integer	Mapped	Questionnaire
	MorseChangeTransfer	Gait assessment (e.g. curve posture, low balance, abnormal, etc.) at change	0 = normal gait/bedrest/immobile 10 = imperfect gait 20 = unsafe gait	Integer	Mapped	Questionnaire
	MorseChangeMental	Mental status assessment at change	0 = conscious of their abilities 15 = unconscious of their abilities	Integer	Mapped	Questionnaire
	MorseChangeTotalScore	Overall Morse score at change	0–125	Integer	Mapped	Questionnaire
10-meter Timed Walking Test (TWT)	TWTInitialDate	TWT execution date at baseline		Date	Mapped	Questionnaire
	TWTInitialTime_m	TWT perfomance time at baseline (mean between two trials)	[s]	Decimal	Mapped	Questionnaire
	TWTInitialSteps_m	TWT perfomance number of steps at baseline (mean between two trials)	[]	Decimal	Mapped	Questionnaire
	TWTDischargeDate	TWT execution date at discharge		Date	Mapped	Questionnaire
	TWTDischargeTime_m	TWT performance time at discharge (mean between two trials)	[s]	Decimal	Mapped	Questionnaire
	TWTDischargeSteps_m	TWT performance number of steps at discharge (mean between two trials)	[]	Decimal	Mapped	Questionnaire
Barthel	BarthelAdmissionDate	Barthel administration date at admission		Date	Mapped	Questionnaire
	BarthelAdmissionHygiene	Need assistance on grooming (i.e. personal care) at admission	0–5	Integer	Mapped	Questionnaire
	BarthelAdmissionWash	Need assistance on bathing at admission	0–5	Integer	Mapped	Questionnaire
	BarthelAdmissionNutrition	Need of assistance in feeding at admission	0–10	Integer	Mapped	Questionnaire
	BarthelAdmissionDress	Need of assistance in dressing at admission	0–10	Integer	Mapped	Questionnaire
	BarthelAdmissionIntestinalincont	Presence or absence of fecal incontinence at admission	0–10	Integer	Mapped	Questionnaire
	BarthelAdmissionUrinaryincont	Presence or absence of urinary incontinence at admission	0–10	Integer	Mapped	Questionnaire
	BarthelAdmissionToilet	Need of assistance in toilet use at admission	0–10	Integer	Mapped	Questionnaire
	BarthelAdmissionTransfer	Need of assistance in transfers (bed to chair and back) at admission	0–15	Integer	Mapped	Questionnaire
	BarthelAdmissionWalk	Need of assistance on mobility (on level surfaces) at admission	0–15	Integer	Mapped	Questionnaire
	BarthelAdmissionStairs	Need of assistance on stairs climbing at admission	0–10	Integer	Mapped	Questionnaire
	BarthelAdmissionWheelchair	Need of assistance in using the wheelchair (if needed) at admission	0–5	Integer	Mapped	Questionnaire
	BarthelAdmissionTotalScore	Barthel total score at admission	0–100	Integer	Mapped	Questionnaire
	BarthelMidDate	Barthel administration date at mid rehab pathway		Date	Mapped	Questionnaire
	BarthelMidHygiene	Need assistance on grooming (i.e. personal care) at mid rehab pathway	0–5	Integer	Mapped	Questionnaire
	BarthelMidWash	Need assistance on bathing at mid rehab pathway	0–5	Integer	Mapped	Questionnaire
	BarthelMidNutrition	Need of assistance in feeding at mid rehab pathway	0–10	Integer	Mapped	Questionnaire
	BarthelMidDress	Need of assistance in dressing at mid rehab pathway	0–10	Integer	Mapped	Questionnaire
	BarthelMidIntestinalincont	Presence or absence of fecal incontinence at mid rehab pathway	0–10	Integer	Mapped	Questionnaire
	BarthelMidUrinaryincont	Presence or absence of urinary incontinence at mid rehab pathway	0–10	Integer	Mapped	Questionnaire
	BarthelMidToilet	Need of assistance in toilet use at mid rehab pathway	0–10	Integer	Mapped	Questionnaire
	BarthelMidTransfer	Need of assistance in transfers (bed to chair and back) at mid rehab pathway	0–15	Integer	Mapped	Questionnaire
	BarthelMidWalk	Need of assistance on mobility (on level surfaces) at mid rehab pathway	0–15	Integer	Mapped	Questionnaire
	BarthelMidStairs	Need of assistance on stairs climbing at mid rehab pathway	0–10	Integer	Mapped	Questionnaire
	BarthelMidWheelchair	Need of assistance in using the wheelchair (if needed) at mid rehab pathway	0–5	Integer	Mapped	Questionnaire
	BarthelMidTotalScore	Barthel total score at mid rehab pathway	0–100	Integer	Mapped	Questionnaire
	BarthelDischargeDate	Barthel administration date at discharge		Date	Mapped	Questionnaire
	BarthelDischargeHygiene	Need assistance on grooming (i.e. personal care) at discharge	0–5	Integer	Mapped	Questionnaire
	BarthelDischargeWash	Need assistance on bathing at discharge	0–5	Integer	Mapped	Questionnaire
	BarthelDischargeNutrition	Need of assistance in feeding at discharge	0–10	Integer	Mapped	Questionnaire
	BarthelDischargeDress	Need of assistance in dressing at discharge	0–10	Integer	Mapped	Questionnaire
	BarthelDischargeIntestinalincont	Presence or absence of fecal incontinence at discharge	0–10	Integer	Mapped	Questionnaire
	BarthelDischargeUrinaryincont	Presence or absence of urinary incontinence at discharge	0–10	Integer	Mapped	Questionnaire
	BarthelDischargeToilet	Need of assistance in toilet use at discharge	0–10	Integer	Mapped	Questionnaire
	BarthelDischargeTransfer	Need of assistance in transfers (bed to chair and back) at discharge	0–15	Integer	Mapped	Questionnaire
	BarthelDischargeWalk	Need of assistance on mobility (on level surfaces) at discharge	0–15	Integer	Mapped	Questionnaire
	BarthelDischargeStairs	Need of assistance on stairs climbing at discharge	0–10	Integer	Mapped	Questionnaire
	BarthelDischargeWheelchair	Need of assistance in using the wheelchair (if needed) at discharge	0–5	Integer	Mapped	Questionnaire
	BarthelDischargeTotalScore	Barthel total score at discharge	0–100	Integer	Mapped	Questionnaire
Emilia-Romagna Region (ERR) Survey	RERMultifAdmissionDate	Emilia-Romagna Region (ERR) questionnaire on fall risk in hospital. Administration date at admission		Date	Mapped	Questionnaire
	RERMultifAdmissionHFall	RER questionnaire at admission. Occurrence of falls in previous year	0 = no, 1 = yes	Categorical	Mapped	Questionnaire
	RERMultifAdmissionFoF	RER questionnaire at admission. Fear of falling	0 = no, 1 = yes	Categorical	Mapped	Questionnaire
	RERMultifAdmissionDrugCardio	RER questionnaire at admission. Use of drugs from the cardiologic area	0 = no, 1 = yes	Categorical	Mapped	Questionnaire
	RERMultifAdmissionDrugPsyco	RER questionnaire at admission. Use of drugs from the psychotropic area	0 = no, 1 = yes	Categorical	Mapped	Questionnaire
	RERMultifAdmissionDrugOtherThree	RER questionnaire at admission. Use of three or more drugs other than those from the cardiologic or psychotropic areas	0 = no, 1 = yes	Categorical	Mapped	Questionnaire
	RERMultifAdmissionDiabetes	RER questionnaire at admission. Diabetes	0 = no, 1 = yes	Categorical	Mapped	Questionnaire
	RERMultifAdmissionCognitiveImp	RER questionnaire at admission. Cognitive impairment	0 = no, 1 = yes	Categorical	Mapped	Questionnaire
	RERMultifDischargeDate	RER questionnaire. Administration date at discharge		Date	Mapped	Questionnaire
	RERMultifDischargeHFall	RER questionnaire at discharge. Occurrence of falls in previous year	0 = no, 1 = yes	Categorical	Mapped	Questionnaire
	RERMultifDischargeFallHS	RER questionnaire at discharge. Occurrence of falls during the hospital stay	0 = no, 1 = yes	Categorical	Mapped	Questionnaire
	RERMultifDischargeFoF	RER questionnaire at discharge. Fear of falling	0 = no, 1 = yes	Categorical	Mapped	Questionnaire
	RERMultifDischargeDrugCardio	RER questionnaire at discharge. Use of drugs from the cardiologic area	0 = no, 1 = yes	Categorical	Mapped	Questionnaire
	RERMultifDischargeDrugPsyco	RER questionnaire at discharge. Use of drugs from the psychotropic area	0 = no, 1 = yes	Categorical	Mapped	Questionnaire
	RERMultifDischargeDrugOtherThree	RER questionnaire at discharge. Use of three or more drugs other than those from the cardiologic or psychotropic areas	0 = no, 1 = yes	Categorical	Mapped	Questionnaire
	RERMultifDischargeDiabetes	RER questionnaire at discharge. Diabetes	0 = no, 1 = yes	Categorical	Mapped	Questionnaire
	RERMultifDischargeCognitiveImp	RER questionnaire at discharge. Cognitive impairment	0 = no, 1 = yes	Categorical	Mapped	Questionnaire

### Standardization in HL7-FHIR

**Fig. 3:**
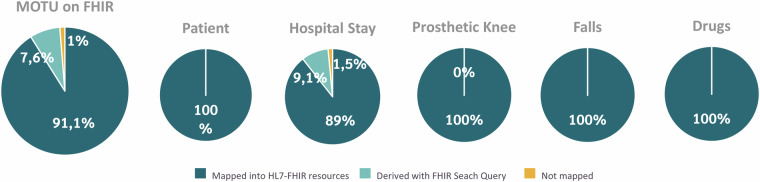
Percentage of standardization into HL7_FHIR of the different original CSV dataset.

**Fig. 4:**
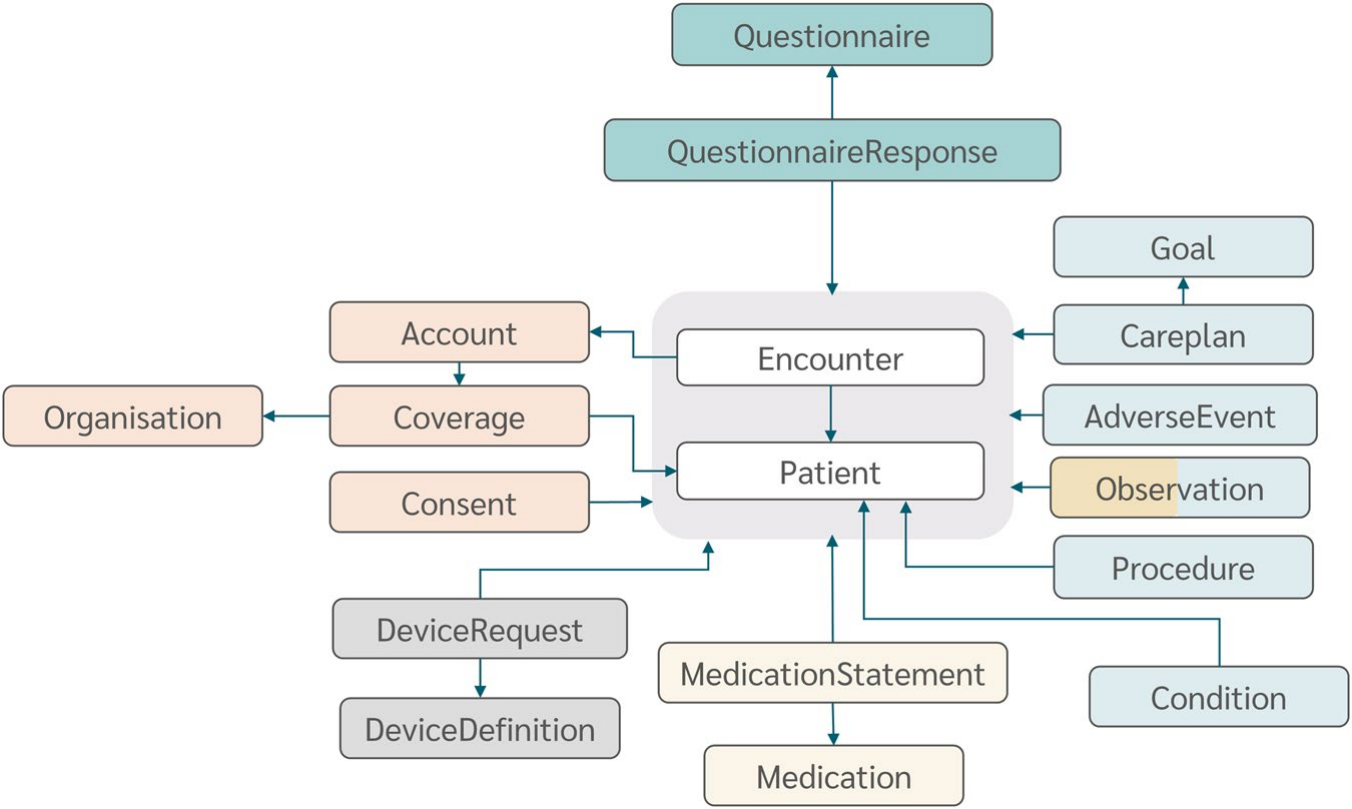
FHIR resources. The color map refers to the different sources of information depicted in Fig. 2. Light blue: clinical evaluations, orange: administrative info, yellow: functional test, blue: questionnaires, grey = prosthetic knee, light yellow = drug prescription.

From the initial set of 157 variables characterizing the CSV dataset, we successfully determined the correspondence with FHIR for 155 variables (98.7% of the dataset). We mapped 143 variables (91.1%) into FHIR resources and 12 variables (1.3%) derivable from FHIR Search Query as shown in Fig. 3. We failed to map two variables (3%) of the overall dataset: HFall and StumpLength in the Hospital Stay table, because it was not possible to identify any ontology associated with any FHIR R4 resource that describes the concept expressed by these variables. The list of variables and the related FHIR mapping is depicted in Table 1. The overall mapping procedure generated 18 distinct resources and 12 FHIR Search Queries. Figure 4 illustrates the relationship among FHIR resources. The fundamental pillar of this dataset revolves around the hospital stay, which is modeled as an Encounter resource with a reference to a Patient resource. The Patient-Encounter couple of aggregated resources is in turn referenced by different resource types, including QuestionnaireResponse, Observation, CarePlan, AdverseEvent, MedicationStatement, DeviceUsage, Account, and Consent. We employed established and widely used dictionaries, namely SNOMED CT^46^, LOINC^47^, the Anatomical Therapeutic Chemical (ATC) classification system^48^, the Unified Code of Measure (UCUM)^49^, the International Classification of Disease 10^th^ edition (ICD-10)^50^, and the NCI Thesaurus (NCIt)^51^. Where a direct linkage with the dictionaries mentioned above was absent, custom code systems were introduced for comprehensive coverage. A total of 2 CodeSystems were generated to represent the following concepts:

1. Motu-encounter-id. This code system delineates the identifier linked to each Encounter resource. Its values denote the unique combination of anonymous patient ID and admission date that identifies each hospital stay.
2. Motu-prosthetic-knee-properties. This code system outlines the technical features of the prosthetic knees (e.g., ManualLock, Polycentric, MPK, etc.) represented through DeviceDefinition resources.

## Technical Validation

### Data check

We checked the distributions of continuous and categorical variables with histograms and tables. We further performed checks on variables representing dates, ensuring consistency between admission and discharge dates and all the dates of tests or questionnaires administered during the hospital stays. Any data inconsistency was solved by confronting paper-based and electronic medical records and applying clinical reasoning. We deleted values of any inconsistency that could not be solved. We performed accuracy and completeness checks on the data collected by students or research assistants.

### FHIR validation

The FHIR validation process encompassed syntactical and semantic validation to ensure the integrity and adherence of the dataset to the FHIR standard. Syntactical validation, performed by Matchbox, rigorously examines the structural correctness of the generated FHIR resources. At the same time, semantic validation, implemented by a dedicated module integrated into the HAPI FHIR server, assesses the resources’ conformity to FHIR profiles. Leveraging the Structure Definitions, which describe the data schema, both levels of validation ensure the compliance of the produced FHIR resources with the specification, realizing a reliable and accurate method for clinical data standardization.

### Comparison with published datasets

An older version of the CSV dataset has already been published^19^, in CSV format only. It consisted of one table with 31 variables about 1486 hospital stays occurred between 2011 and 2017. The dataset presented here consists of five tables with a total of 157 variables. It covers 1962 hospital stays occurred between 2011 and 2020 In addition, we make it available in the FHIR standard format.

A dataset on the persons with lower limb amputation can be found in the publication of Hood and colleagues^18^. This dataset consists of full-body biomechanics data acquired with a motion capture system and demographic and clinical data. Despite the innovative impact of such a dataset, it collects information on a small group of patients (18) and does not provide insights on clinical aspects, adverse events, or technical features on prosthetic devices. To the best of our knowledge, there is no public dataset on people with lower-limb amputation followed in their clinical pathway. The present dataset can be exploited for a variety of purposes, such as improving the personalization of prosthetic choice based on several personal and health-related characteristics or better understanding possible relationships between falls and prosthetic knees.

### Dataset enquiry

We applied two distinct methodologies to query the dataset, utilizing both the CSV and FHIR formats. This approach aimed to validate the consistency of information resulting from the conversion between CSV format and the FHIR resource representation by comparing the number of instances obtained by the two data sources. In querying the CSV format, we utilized Python programming language. On the other hand, for the FHIR resource, we converted the dataset into a Resource Description Framework (RDF) graph, employing SPARQL as the default querying language for data represented in RDF^52^. In selecting queries, we leverage the expertise of two co-authors (P.R., A.D.), who are clinicians associated with the INAIL Centre. They identified five different queries recognized as clinically meaningful. Table 3 provides a concise summary of the queries and their corresponding results.

**Table 3:** Number of instances for five different queries on CSV and FHIR datasets.

Query	Results	
	Python < --CSV	SPARQL < --FHIR
Q1. How many patients who came for an initial supply return for a second hospitalization?	85	85
Q2. How many patients transition from the initial supply of a mechanical knee to a second supply of an electronic knee?	34	34
Q3. How many patients use anxiolytics/antidepressants (ATC Code: N06A and N05A) related to the risk of falls?	67	67
Q4. Hospital stays from patients over 65 years old	674	674
Q5. Which type of knee (i.e., AMK, FK, LF, MPK) were worn by patients who experienced a fall?	AMK - 11	AMK - 11
	FK - 79	FK - 79
	LK - 26	LK - 26
	MPK - 28	MPK - 28

## Usage Notes

For the FHIRed dataset, interested parties and researchers can download an NDJSON formatted version and import it into an FHIR Server through the Bulk Data API. The repository’s documentation section provides more detailed instructions on utilizing the dataset.

The MOTU dataset is valuable for clinicians and researchers focusing on the rehabilitation pathway for lower limb amputation. Its richness encompasses a broad spectrum of information, including clinical, administrative, drug-related, prosthetic knee details, and functional mobility test assessments, among other parameters. It enables a comprehensive analysis of this particular patient group. Moreover, since it is machine-readable, automatic processing pipelines can be enabled by expert engineers employing FHIR data standard.

Although there are no official statistics, we expect that in Italy, during the years 2011–2020, the vast majority of people with trans-femoral amputation for work-related injuries chose the INAIL Prosthesis Center for rehabilitation training, as it was the only one offering this service. Contrariwise, only a minor fraction of non-work-related trans-femoral amputees have undergone rehabilitation at this center. Since work-related amputees are over-represented, the MOTU dataset as such cannot be considered representative of the whole Italian population of trans-femoral amputees. However, separate analyses on these two subpopulations can be done using the information on the third-party payer (CSV dataset: table HospitalStay, variable ThirdPayer; FHIRed dataset: field Account.Coverage (pointing to the Coverage resource)). Hospital stays for work-related amputations can be unambiguously identified as those subsidized by INAIL, while for the others the payer is annotated to be either the Local Health Service (ASL) or private (Table 1).

The time from amputation to receipt of the first prosthesis, which is pivotal to address numerous clinical questions, can be derived from the amputation date (CSV dataset: table HospitalStay, variable AmputationDate; FHIRed dataset: field Procedure.occurrence_x_) and the admission date (CSV dataset: table HospitalStay, variable AdmissionDate; FHIRed dataset: field Encounter.dtartDate) for first prosthetic fitting (CSV dataset: table HospitalStay, variable FirstdeliveryRenewal=“FirstDeliv”; FHIRed dataset: those instances with empty Encounter.hospitalization.reAdmission field).

The number of comorbidities is available in tableHospitalStay, variable NComorbidities. It was estimated from the number of fields filled with pathological annotations in the electronic health record section dedicated to the physical examination^20^. Other direct or indirect information about the medical comorbidities of the patients can be found in the Morse Scale (CSV dataset: table HospitalStay, variables Morse[Admission/Discharge/Change]Pathologies; FHIRed dataset: Morse Questionnaire resource), in the ERR survey (CSV dataset: table HospitalStay, variableRERMultif[Admission/Discharge]Diabetes; FHIRed dataset: ERR Questionnaire resource); in a dedicated variable about diabetes (CSV dataset: table HospitalStay, variable Diabetes; FHIRed dataset: Condition resource), in the Barthel Index (CSV dataset: table HospitalStay, variables Barthel[Admission/Discharge/Mid]Intestinalincont and Barthel[Admission/Discharge/Mid]Urinaryincont; FHIRed dataset: Barthel Questionnaire resource), and in the list of all drugs taken by the patients during the hospital stay (CSV dataset: table Drug; FHIRed dataset: Medication resource).

## Data Availability

“The MOTU-to-FHIR Mapping Pipeline code is accessible on GitLab at https://gitlab.com/almahealthdb/ahdb-mapping-service/. This repository contains all necessary scripts, configuration files, and instructions for transforming the MOTU CSV dataset into the FHIRed dataset based on the HL7 FHIR standard (R4 version). It also provides a Docker Compose file that describes the configuration of Matchbox and the HAPI FHIR Server, facilitating automated deployment of the pipeline. For detailed instructions on running the pipeline, please refer to the README.md file in the repository”.
